# Impact of grape polyphenols on *Akkermansia muciniphila* and the gut barrier

**DOI:** 10.3934/microbiol.2022035

**Published:** 2022-12-22

**Authors:** Esther Mezhibovsky, Yue Wu, Fiona G. Bawagan, Kevin M. Tveter, Samantha Szeto, Diana Roopchand

**Affiliations:** 1 Rutgers, The State University of New Jersey, Department of Food Science, Institute for Food Nutrition and Health [Center for Microbiome, Nutrition and Health & Rutgers Center for Lipid Research], 61 Dudley Road, New Brunswick, NJ 08901, USA; 2 Rutgers, The State University of New Jersey, Department of Nutritional Sciences Graduate Program, New Brunswick, NJ 08901, USA

**Keywords:** polyphenols, proanthocyanidin, dietary fat, *Akkermansia muciniphila*, gut microbiota, mucus layer, gut barrier, inflammation

## Abstract

A healthy gastrointestinal tract functions as a highly selective barrier, allowing the absorption of nutrients and metabolites while preventing gut bacteria and other xenobiotic compounds from entering host circulation and tissues. The intestinal epithelium and intestinal mucus provide a physical first line of defense against resident microbes, pathogens and xenotoxic compounds. Prior studies have indicated that the gut microbe *Akkermansia muciniphila*, a mucin-metabolizer, can stimulate intestinal mucin thickness to improve gut barrier integrity. Grape polyphenol (GP) extracts rich in B-type proanthocyanidin (PAC) compounds have been found to increase the relative abundance of *A. muciniphila*, suggesting that PACs alter the gut microbiota to support a healthy gut barrier. To further investigate the effect of GPs on the gut barrier and *A. muciniphila*, male C57BL/6 mice were fed a high-fat diet (HFD) or low-fat diet (LFD) with or without 1% GPs (HFD-GP, LFD-GP) for 12 weeks. Compared to the mice fed unsupplemented diets, GP-supplemented mice showed increased relative abundance of fecal and cecal *A. muciniphila*, a reduction in total bacteria, a diminished colon mucus layer and increased fecal mucus content. GP supplementation also reduced the presence of goblet cells regardless of dietary fat. Compared to the HFD group, ileal gene expression of lipopolysaccharide (LPS)-binding protein (*Lbp*), an acute-phase protein that promotes pro-inflammatory cytokine expression, was reduced in the HFD-GP group, suggesting reduced LPS in circulation. Despite depletion of the colonic mucus layer, markers of inflammation (*Ifng, Il1b, Tnfa, and Nos2*) were similar among the four groups, with the exception that ileal *Il6* mRNA levels were lower in the LFD-GP group compared to the LFD group. Our findings suggest that the GP-induced increase in *A. muciniphila* promotes redistribution of the intestinal mucus layer to the intestinal lumen, and that the GP-induced decrease in total bacteria results in a less inflammatory intestinal milieu.

**Figure:**
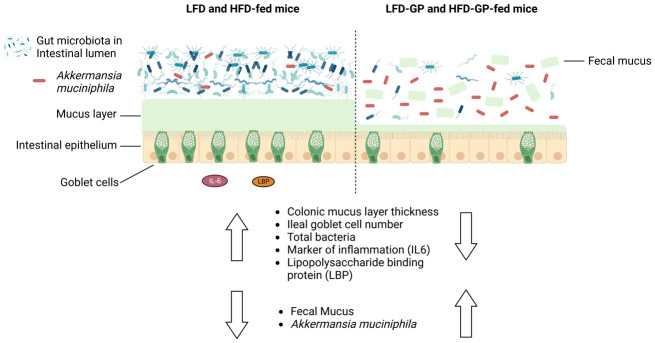

## 1. Introduction

Components of the gut barrier include the intestinal epithelium cells, intestinal mucus layer and secreted antimicrobial peptides/enzymes [1],[2]. Resident commensal gut bacteria also contribute to the gut barrier via the production of beneficial metabolites or prevention of the overgrowth of pathogenic bacteria [3]. The gel-like mucus present in the small and large intestines is predominantly composed of mucin 2 (Muc2), a glycoprotein released by goblet cells of the intestinal epithelium. Mucus serves to lubricate surfaces of the gastrointestinal tract, facilitate excretion of fecal matter and provide a physical and functional barrier between host and luminal gut bacteria [1],[2]. The overall protective role of the intestinal mucus layer was demonstrated in Muc2 knockout mice (Muc2^−/−^), which were shown to develop more severe colitis than wild-type mice [4] and have increased susceptibility to pathogenic *Escherichia coli* infections [5]. The small intestine has a single discontinuous mucus layer to facilitate nutrient absorption [6]. Microscopic examination of longitudinal sections of the colon revealed that mucus organization depends on the colon content [7]. In the proximal colon where digesta are more liquid, a significant mucus layer was not observed [7]. Outer and inner layers of mucus have been described in the distal colon [6], but this organization may vary depending on transverse or longitudinal sectioning of the tissue and the absence or presence of a fecal pellet [6],[7]. The loose outer mucus layer may be colonized and metabolized by some species, such as *Akkermansia muciniphila* [8]. The inner colonic mucus adjacent to the intestinal epithelium was reported to be sterile [9], but other studies have shown that some species of bacteria can penetrate the inner mucus layer to associate with colonic crypts, not just in disease states, but also in healthy symbiotic relationships [10]–[14].

Diet is a major regulator of the gut microbiota and intestinal barrier. For example, certain spices, amino acids (tryptophan, L-alanine) or fatty acids (capric acid, lauric acid, linolenic acid) have been shown to reduce tight junction protein (TJP) expression and increase gut permeability, while other amino acids (glutamine, casein peptides), minerals (zinc) or polyphenols (epigallocatechin gallate (EGCG), isoflavones) have increased TJP expression and reduced gut permeability [15]. High-fat diet (HFD) feeding promotes gut microbial dysbiosis, mucus layer thinning, altered expression of TJPs and increased synthesis of hydrophobic bile acids, which are all factors that contribute to a more permeable gut barrier [16]–[19]. An HFD induces metabolic endotoxemia, which is the translocation of bacteria and proinflammatory bacterial components, such as lipopolysaccharide (LPS), across the gut barrier into circulation [16],[20],[21]. LPS initially interacts with LPS-binding proteins (LBPs) and CD14, which are accessory molecules that facilitate the transfer of LPS to toll-like receptor-4 (TLR4)-MD2 complexes that stimulate the host's innate immune response [22]. Proinflammatory cytokines such as interleukin 6 (IL-6), IL-1β, tumor necrosis factor α (TNFα) and interferon ƴ (IFNƴ), as well as inducible nitric oxide synthase (iNOS), defend the host from pathogens and promote tissue healing by recruiting other immune cells [23]. iNOS combines superoxide with NO to form reactive oxidative species (ROS) with bactericidal activity [24]. While transient induction of ROS and inflammation are critical to intestinal homeostasis, HFD feeding leads to chronic low-grade inflammation and disease [25].

Special interest has been placed on *A. muciniphila*, a mucin-metabolizing species of phylum Verrucomicrobiota [8], due to its frequent association with improved cardiometabolic outcomes and gut barrier integrity [26]–[37], with some exceptions [38],[39]. HFD-fed mice that were orally administered viable *A. muciniphila* showed increased colon mucus layer thickness and less LPS in circulation, suggesting protection from metabolic endotoxemia [26]. Intestinal organoids exposed to a growth medium containing *A. muciniphila* metabolites had altered expression of genes involved in cellular lipid metabolism and growth [40], providing evidence that *A. muciniphila* metabolites can influence host metabolism. Subjects who were overweight and obese showed metabolic improvements after oral supplementation with either live or pasteurized *A. muciniphila* [34]. Purified Amuc_1100, an outer membrane protein of *A. muciniphila*, was shown to improve metabolic outcomes in obese mice, modulate cytokine expression in vitro and increase transepithelial electrical resistance, a measure of gut barrier function [28],[41].

Our previous studies have shown that mice fed an HFD or LFD supplemented with proanthocyanidin (PAC)-rich grape polyphenols (GPs) develop a reproducible bloom of *A. muciniphila* and significantly reduced abundance of genera within the Firmicutes phylum [27],[42]–[44]. Other rodent or human studies of polyphenol supplementation have also reported increased relative abundance of *A. muciniphila* in association with improved metabolic phenotypes [32],[33],[45]–[47]. GP-supplementation reduced diet-induced weight gain independently from food intake [27],[42] and caloric absorption [48]; therefore, other mechanisms must contribute to metabolic improvements. PACs, the most abundant polyphenols in our GP extract, have low intestinal absorption and alter the gut microbial community, suggesting direct effects within the intestinal milieu [49]. Herein, mice were fed an HFD or LFD with or without GPs to further investigate the consequences of the GP-induced *A. muciniphila* bloom on intestinal morphology, as well as markers of gut inflammation, permeability and mucin production.

## 2. Materials and methods

### 2.1. Diets

GPs were extracted from Concord grape pomace (kindly provided by Welch Foods, Inc., Concord, MA, USA) as previously described for the preparation of GPs complexed with soy protein isolate (GP-SPI) [42]. This prior study included the biochemical characterization of the same batch of GP extract used in the present study and consisted of a mixture of catechin/epicatechin monomers and PAC B-type dimers, followed by trimers, dimer gallates, trimer gallates, tetramers and pentamers [42]. The nutritional composition of GP-SPI and SPI ingredients were determined by Medallion Laboratories (Minneapolis, MN, USA) in accordance with the Association of Official Agricultural Chemists (AOAC) methods for ash, moisture, proteins, dietary ﬁber and total fat; the total carbohydrates were determined by difference (100%-moisture–ash–protein-total fat), and calories by calculation (Supplementary Table 1) [42]. Nutritional analysis data were used to formulate HFDs and LFDs containing soy protein isolate (SPI) alone or GP-SPI (Research Diets, New Brunswick, NJ, USA) with defined nutritional composition (Supplementary Table 2), which have been previously described [42]. Starting at age 5 weeks, mice were fed the following ingredient-matched diet formulations: 1) HFD containing 10% SPI (5.91 kCal/g; HFD); 2) HFD formulated with 10% GP-SPI, delivering 1% GP (5.59 kCal/g; HFD-GP); 3) LFD containing 10% SPI (4.51 kCal/g; LFD); and 4) LFD formulated with 10% GP-SPI, delivering 1% GP (4.51 kCal/g; LFD-GP).

### 2.2. Mice

Five-week-old male C57BL/6J mice (n = 20, Jackson Laboratories, Bar Harbor, Maine, USA) were acclimated on an LFD for 1 week. Oral glucose tolerance (OGT) tests were performed at baseline after acclimation. OGT data were used to divide mice into four diet groups (n = 5 per group) with similar baseline OGTs.

Fecal samples were collected on day 2, week 2 and week 4 of the diet intervention. At week 12 of intervention, mice were euthanized by carbon dioxide asphyxiation, followed by cardiac puncture and collection of tissue, as previously described [27]. Sections of ileal and colon tissue with fecal content were fixed with methyl carnoy (60% (v/v) methanol, 30% v/v chloroform, 10% (v/v) glacial acetic acid), for 24 hours to preserve the mucus layer integrity for downstream staining, as previously described [50].

### 2.3. Bacterial qPCR

Fecal and cecal gDNA were extracted from fecal samples collected on day 2, week 2 and week 4, and cecal content was collected at sacrifice during week 12 of the diet intervention, according to the manufacturer's instructions (DNeasy PowerSoil Pro Kit, QIAGEN, Germantown, MD, USA). Additionally, 30–40 mg of frozen fecal samples were homogenized for 10 min by using a 1:3 ratio of glass beads in a GenoGrinder (GenoGrinder 1600 MiniG, SPEX Sample Prep, Metuchen, NJ, USA). After extraction, DNA concentrations were quantified by using a Nanodrop system (Thermo Fisher Scientific, Inc., Waltham, Massachusetts, USA) and then diluted to 2.5 ng/µL in sterile water. Quanitative PCR (qPCR), by utilizing Power SYBR Green PCR Master Mix (Applied Biosystems, Carlsbad, CA), was conducted for the quantitative analysis of microbial DNA. *Akkermansia muciniphila* was quantified by using species-specific primers (AM1: 5′-CAGCACGTGAAGGTGGGGAC-3′; AM2: 5′-CCTTGCGGTTGGCTTCAGAT-3′). Total microbial DNA was quantified using universal bacterial primers (U341F: 5′-CCTACGGGRSGCAGCAG-3′ and U515R: 5′-TACCGCGGCKGCTGRCAC-3′). Standard curves for both *A. muciniphila* and universal bacteria were created by using serial dilutions of pure culture *A. muciniphila* gDNA (2, 0.4, 0.08, 0.016, 0.0032, 0.00064, 0.000128 ng/µL). The abundance was determined by adjusting the concentrations of microbial DNA determined by qPCR for the dilutions performed during DNA extraction (1:50), normalization (dilution to 2.5 ng/µL) and qPCR set-up (1:6). Relative abundance of *A. muciniphila* was calculated by dividing by concentration of total bacteria.

### 2.4. Tissue qPCR

Ileum and colon tissues (30–50 mg) were homogenized with three 2.5 mm stainless steel beads and Qiazol reagent by using GenoGrinder 1600 MiniG (SPEX Sample Prep, Metuchen, NJ, USA), and RNA was isolated by using RNeasy Plus Universal Mini Kit (QIAGEN, Germantown, MD, USA). Extracted RNA was quantified by using a Nanodrop system (Thermo Fisher Scientific, Inc., Waltham, Massachusetts, USA). RT-qPCR was performed on a SimpliAmp Thermal Cycler (Applied Biosystems by Thermo Fisher Scientific) by using a high-capacity cDNA reverse transcription kit to reverse-transcribe 5 µg of mRNA to 25 µL of cDNA, diluting with 50 µL of RNAase free water. qPCR was performed by using a qPCR machine (QuantStudio 3 Real-Time PCR Instrument 96-well 0.1 mL Block, Applied Biosystem by Thermo Fisher Scientific). Thermo Fisher TaqMan primer sets, i.e., IFN-γ (Mm01168134_m1), IL-6 (Mm00446190_m1), IL-1β (Mm00434228_m1), iNOS (Mm00440502_m1), TNFα (Mm00443258_m1), LBP (Mm00493139_m1), occludin (Ocln; Mm00500912_m1), TJP 1 (TJP1; Mm00493699_m1), mucin 2 (Mm01276696_m1) and mucin 3 (Mm01207064_m1) were used as biomarkers for intestinal inflammation, permeability and mucin production. Additionally, 18s rRNA (Mm03928990_g1) was used as the housekeeping gene. TaqMan Fast Universal PCR Master Mix (Cat# 4366072, Thermo Fisher) was used for qPCR according to the manufacturer's protocol: a melting temperature of 20 s at 95 °C, followed by 40 cycles of two-step PCR denaturation at 95 °C for 1 min and annealing extension at 60 °C for 20 s at a cooling rate of 2.12 °C/s. Duplicated samples containing 2 µL of cDNA and 8 µL of MasterMix were plated on 96-well plates. Means of the duplicates were taken, and the relative expressions of target genes were normalized with the housekeeping gene.

### 2.5. Alcian blue and periodic acid schiff, immunofluorescent staining and hematoxylin & eosin staining and quantification of intestinal cross sections

Methyl-carnoy-fixed ileal and colonic intestinal tissue sections with digesta were embedded in paraffin and sectioned into 5 µm cross-sections. Ileal and colonic sections were stained by hematoxylin & eosin (H&E) staining (Rutgers Research Pathology Services, Piscataway, NJ, USA) and used to investigate changes to intestinal morphology, specifically ileal villus length, crypt depth and number, and colon crypt depth and crypt number. Alcian blue and periodic acid schiff (AB/PAS) and immunofluorescent (IF) staining was performed to quantify intestinal mucus thickness and goblet cell number. For AB/PAS and IF staining, sections were dewaxed with xylene substitute (Sigma-Aldrich, Saint Louis, Missouri, USA) for 10 min at 60 °C, and again at room temperature. Slides were rehydrated with an ethanol gradient (95, 70, 50, 30%) for 2 min at each concentration, and then with de-ionized water, prior to staining.

An AB (pH 2.5)/PAS stain kit (Cat# 87023 Thermo Scientific ™ Richard-Allan scientific ™) was used to stain intestinal mucus and quantify the thickness of the mucus layer and goblet cell number. At pH 2.5, AB stains carboxylated and sulfatic mucopolysaccharides a blue color. PAS stains by reacting with the free aldehyde groups within monosaccharide units of the mucins with Schiff reagent to form a bright red magenta product. AB/PAS detects both neutral and acidic mucins, and staining together captures most mucin types within the intestinal cross section. AB/PAS staining was performed according to the kit protocol. Post-staining, all sections were dehydrated with an increasing ethanol concentration gradient (95, 100%) for 1 min at each concentration and then cleared with xylene for 2 min before mounting them with ProLong™ Gold Antifade Mountant (Cat # P36930).

Anti-Muc2 IF staining was performed as described previously [50] to quantify the thickness of the intestinal mucus layer. After dewaxing and rehydrating, a heat-mediated antigen retrieval was performed in 10 mM citrate buffer (pH 6.0) by using an Oster steamer for 45 min, after which the tissue sections were blocked with 10% normal goat serum (Cat# PCN5000, Fisher Scientific) in 1X phosphate buffered saline (PBS) solution for 1 hour; they were then cooled for 20 min and washed with 1X PBS for 5 min. Intestinal Muc2 was labeled with 1 µg/mL of rabbit anti-Muc2 (Cat# RP1038 now A01212, Boster Bio, Pleasanton, CA, USA) prepared in 10% goat serum in 1X PBS by incubating mounted sections overnight in a humidity chamber (VWR Cat# 76278-848) at 4 °C, followed by three washes with 1X PBS. Goat anti-rabbit secondary antibody conjugated to AlexaFluor® 488 (green) at 0.5 µg/mL of dilution (Cat# A11008, Life Technologies) in 10% goat serum in 1X PBS was applied for 2 hours in a humidity chamber at room temperature, after which, slides were washed three times with 1X PBS. Slides were air-dried for 2 min before mounting them with ProLong™ Gold Antifade Mountant (Cat # P36930).

Images were taken by using an Olympus FSX100 microscope (Waltham, Massachusetts, USA). To prevent biased selection of regions in intestinal cross sections, flags were placed equidistantly on 4× images along the intestinal epithelium, and 14× images were taken at these randomly selected areas. Ten images were taken per cross section at a 14× magnification. Length measurements and object counting were performed by using NIH ImageJ1 (National Institutes of Health, Bethesda, MD, USA). A line was drawn over the image scale by using the ‘line selection tool’, and the ‘Set Scale’ window was set to the known micrometer distance to establish the pixel-to-length relationship for all length measurements. The ‘point tool’ was used for counting objects. Quantification of the mucus thickness and intestinal morphology was done by two independent research assistants to ensure reproducible data.

The colonic mucus thickness was measured from IF and AB/PAS staining by using the ‘line selection tool’. Ten regions per 14× image were selected for mucus thickness measurement, totaling 100 measurements per mouse. See Supplementary Figure 1A for an image displaying how mucus thickness was measured. Mucus thickness is presented as thickness ± standard deviation (SD; µm) for each mouse.

The ileal goblet cell number was quantified from AB/PAS-stained images obtained at 14× magnification by using the ‘point tool’. Goblet cells were quantified from villus and crypts separately. Only intact villi and villi fully within the image plane were used for goblet cell quantification. See Supplementary Figure 1 for an image indicating quantified goblet cells on villi and in crypts. Data are presented as goblet cell number per ileal villus ± SD and goblet cell number per ileal crypt for each mouse. The colonic and ileal crypt depth and ileal villus length were measured by using the ‘line selection tool’. Only intact villi and crypts and those within the image plane were used for quantification. See Supplementary Figure 1 for an image displaying an example of crypt and ileal depth measurement. Crypt and villus number were quantified by using the ‘point tool’.

Crypt and villi number per cross sections were obtained from H&E stains. Ileal and colonic images were first stitched in Microsoft PowerPoint to get entire cross sections. Colon and ileal crypts and ileal villi were quantified from entire cross sections by using the ‘point tool’. Only villi and crypts within the image plane were quantified. See Supplementary Figure 1 for an image displaying counted crypts and villi, and Supplementary Figures 2 and 3 for representations of the ileal and colon cross sections used.

### 2.6. Mucus detection in colon content and fecal samples

A fecal mucin assay kit (Cat. # CSR-FFA-MU-K01E, Cosmo Bio Co., Ltd.) was used for fluorometric detection and quantification of the mucin in feces and colon content. Fecal samples from weeks 2 and 4 and colon content collected during sacrifice at week 12 were freeze-dried overnight in a lyophilizer (Labconco, Kansas City, MO, USA). Mucins were extracted by following the manufacturer's protocol and Bovee-Oudenhoven et al. [51], and fluorescence was measured with a multimode plate reader (CLARIOstar, BMG Labtech, Cary, North Carolina, USA). Mucin content per gram of feces or colon content was calculated against a standard curve of mucin.

### 2.7. Alanine transaminase assay

Alanine transaminase (ALT) assay was performed on cardiac serum collected at week 12 by using a Liquid ALT (SGPT) reagent set (catalog#, A7526-450 Pointe Scientific Inc., Thermo Fisher) per the manufacturer's protocol. Sample absorbance was detected by using a ClarioStar spectrophotometer.

### 2.8. Statistics

Analyses were conducted and graphed by using Prism 9 (GraphPad Software, La Jolla, CA, USA). Normality of the data was confirmed with Shapiro-Wilk's testing. A ROUT test (Q = 1%) was performed to detect outliers on all data sets. Gut phenotypes and qPCR results were analyzed via two-way ANOVA, followed by Holm-Sidak's multiple-comparison test. Pearson correlation analysis was performed to detect significant correlations between *A. muciniphila* relative abundance and gut phenotypes. P < 0.05 was considered significant.

### 2.9. Ethics approval of research

The animal study protocol was approved by Rutgers University IACUC. Protocol 16-030, approval period September 14, 2016 to September 13, 2019.

## 3. Results

### 3.1. GP supplementation increased relative abundance of A. muciniphila

**Figure 1.**
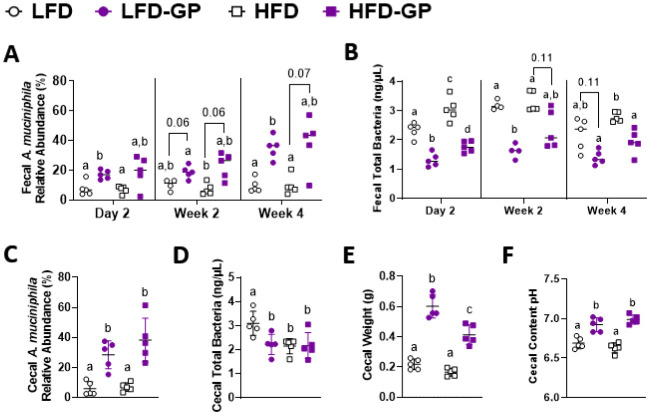
Changes to *A. muciniphila* relative abundance and total bacterial load in feces and cecal content. (A) *A. muciniphila* relative abundance in feces and (B) total bacterial load in feces after 2 days, 2 weeks and 4 weeks of diet intervention, (C) *A. muciniphila* relative abundance in cecal content, (D) total bacterial load in cecal content, (E) whole cecal weight at endpoint and (F) cecal content pH after 12 weeks on diets; (n = 5/diet group). Statistical significance between four groups of parametric data was determined via two-way ANOVA, followed by Holm-Sidak's multiple-comparisons test. Different letters denote statistical significance. p < 0.05 was considered significant. Each symbol denotes a single animal.

Compared to unsupplemented LFD-fed mice, the relative abundance of *A. muciniphila* was significantly increased in mice fed LFD-GP after 2 days, non-significantly increased after 2 weeks (p = 0.06) and again significantly increased after 4 weeks of supplementation (Figure 1A). HFD-GP-fed mice had a trend of increased *A. muciniphila* after 2 (p = 0.06) and 4 weeks (p = 0.07) compared to the HFD-fed mice (Figure 1A). *A. muciniphila* levels did not differ between LFD and HFD or between LFD-GP and HFD-GP groups, indicating that dietary fat did not significantly influence the bloom of this microbe (Figure 1A). At two of three of the evaluated time points, GP supplementation significantly decreased total fecal bacterial load in mice fed LFD-GP and HFD-GP; one of three time points showed a trend of decreased bacterial load (Figure 1B). At week 12, the relative abundances of *A. muciniphila* in cecal content from LFD-GP and HFD-GP groups were 29% and 39%, respectively, which were significantly higher than those of the unsupplemented groups, where relative abundances were between 6–7% (Figure 1C). Compared to LFD-fed mice, the LFD-GP group showed reduced total bacterial load in cecal content, while HFD and HFD-GP groups showed similar levels of total cecal bacteria (Figure 1D). GPs may have a selective antimicrobial effect on murine gut microbes depending on dietary fat exposure and location within the intestinal tract. As previously reported [27],[44], GP-supplementation significantly increased cecal mass (Figure 1E). Increased cecal mass may be due to reduced numbers of bacteria available for metabolism of macronutrients and increased luminal water content, an effect also observed after antibiotic treatment [52]. The optimal pH for *A. muciniphila* growth is 6.5 [53]. Compared to the pH of the LFD (6.69) and HFD (6.63) groups, cecal pH was significantly increased in GP-supplemented mice to a more neutral range of 6.92–6.99 (Figure 1F) and may serve to offset the antimicrobial effect of GPs.

### 3.2. GP-supplemented mice showed reduced colonic mucus layer and redistribution of mucins within intestinal lumen

**Figure 2.**
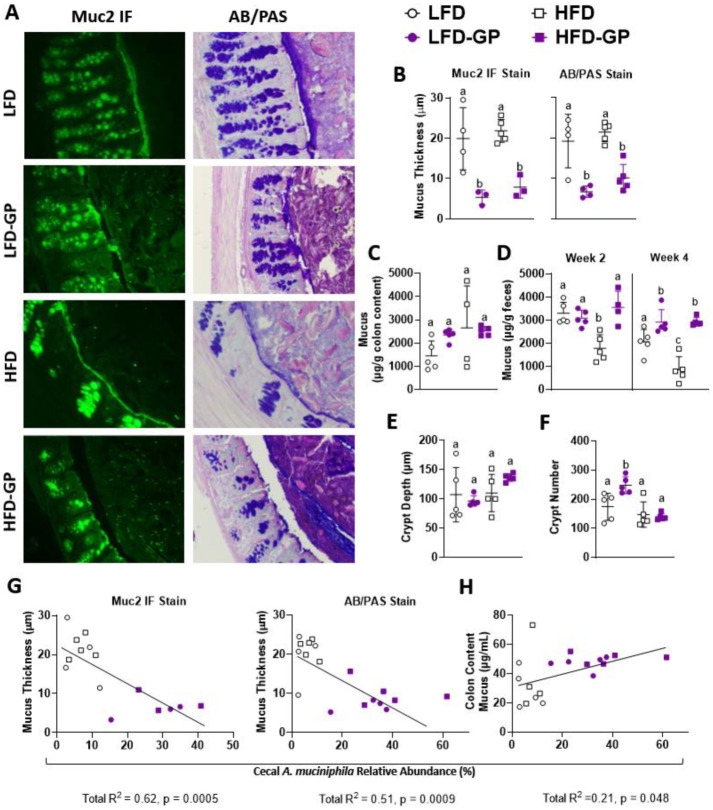
GPs altered colonic mucus layer. (A) Representative images (one mouse selected from n = 3–5 mice/group) of colonic Muc2 IF (green) and AB/PAS stains (pink, purple) at 14× magnification at week 12 to visualize mucus thickness, with (B) quantified mucus thickness from both stains. (C) Mucus content in colonic content at week 12, and (D) fecal pellets at week 2 and week 4 of the diet intervention. (E) Average colonic crypt depth and (F) crypt number per cross section, as quantified from H&E-stained slides. Pearson correlation showing cecal *A. muciniphila* relative abundance verses (G) mucus thickness as measured from IF staining and AB/PAS stain, and verses (H) colon content mucus concentration. Statistical significance between four groups of parametric data was determined via two-way ANOVA, followed by Holm-Sidak's multiple-comparisons test. Different letters note significantly different values. p < 0.05 was considered significant. Each symbol denotes a single animal.

LFD and HFD-fed groups of mice had a visible continuous mucus layer along the colonic epithelium; however, the mucus layer in GP-supplemented mice appeared thinner or absent in anti-Muc2 IF stains and AB/PAS stains (Figure 2A; see Supplementary Figure 2 for representative images of whole colonic cross sections). Mucus thickness measured from anti-Muc2 IF or AB/PAS-stained specimens showed a thinner colonic mucus layer in GP-supplemented mice regardless of dietary fat (Figure 2B). In GP-supplemented mice, anti-Muc2 IF showed numerous rod-like green specks throughout the fecal pellet, which were scarcely present in the LFD or HFD groups when viewed at 14× magnification (Figure 2A). In AB/PAS-stained sections from GP-supplemented mice, the greater abundance of fecal mucus appeared as dark purple staining within the intestinal lumen due to chemical reactions with free aldehydes on polysaccharide moieties of mucins present within the fecal pellet (Figure 2A). The colon images from GP-supplemented mice suggest that the mucus layer that normally appears close to the intestinal epithelium was digested and dispersed throughout the fecal pellet.

The mucus concentration measured in colon content was similar among all diet groups (Figure 2C). Compared to the mice fed a LFD or LFD-GP, fecal mucus content was decreased in the HFD group after 2 weeks; however, mice fed HFD-GP had increased levels, similar to the LFD and LFD-GP groups (Figure 2D). After 4 weeks of intervention, HFD-fed mice still had decreased fecal mucus excretion, while GP supplementation increased fecal mucus content regardless of dietary fat intake (Figure 2D). It is unclear if colonic mucus production was affected due to changes in numbers of goblet cells, as cells were too dense for accurate quantification.

Crypts of the intestinal epithelium harbor stem cells at their base, and progenitor cells travel up the flanks as they mature into differentiated cells found in the upper region of the crypts [54]. Thus, crypt homeostasis is crucial for cell regeneration, cell turnover and adaptations to the intestinal environment. Crypt loss disrupts intestinal epithelium integrity. In dextran sulphate sodium-induced colitis, rodents have been shown to exhibit increased apoptosis and crypt atrophy compared to controls [55]. Additionally, a HFD has been implicated in shortened crypt depth as compared to lower fat diets [56],[57]. Colon crypt depth was similar in all diet groups, as determined by quantitative analysis of colonic cross sections stained by H&E (Figure 2E). The crypt numbers in the small and large intestines are not stagnant, though the rate of crypt fission, i.e., the process of crypt division, occurs at a slower rate in adulthood [58]. The implications of crypt fission are not entirely understood, though there are reports of observing increased fission in patients with obesity after Roux-en-Y gastric bypass [59]; therefore, fission may be an adaptation to altered intestinal surface area and nutrient absorption. Compared to the LFD-fed mice, the LFD-GP group had increased colon crypt numbers, while no changes in colon crypt number were observed between the HFD and HFD-GP groups (Figure 2F).

Correlation analysis of the unsupplemented and GP supplemented groups revealed a significant inverse relationship between the relative abundance of cecal *A. muciniphila* and colonic mucus thickness, as measured by both IF and AB/PAS staining (Figure 2G), and a positive relationship between cecal *A. muciniphila* relative abundance and colon mucus content (Figure 2H). Overall, these data suggest that the GP-induced increase in *A. muciniphila* (Figure 1A and C) led to increased digestion of the colonic mucus layer, regardless of dietary fat, resulting in the redistribution of mucus throughout the fecal pellet.

### 3.3. GP supplementation reduces the number of goblet cells in the ileum

**Figure 3.**
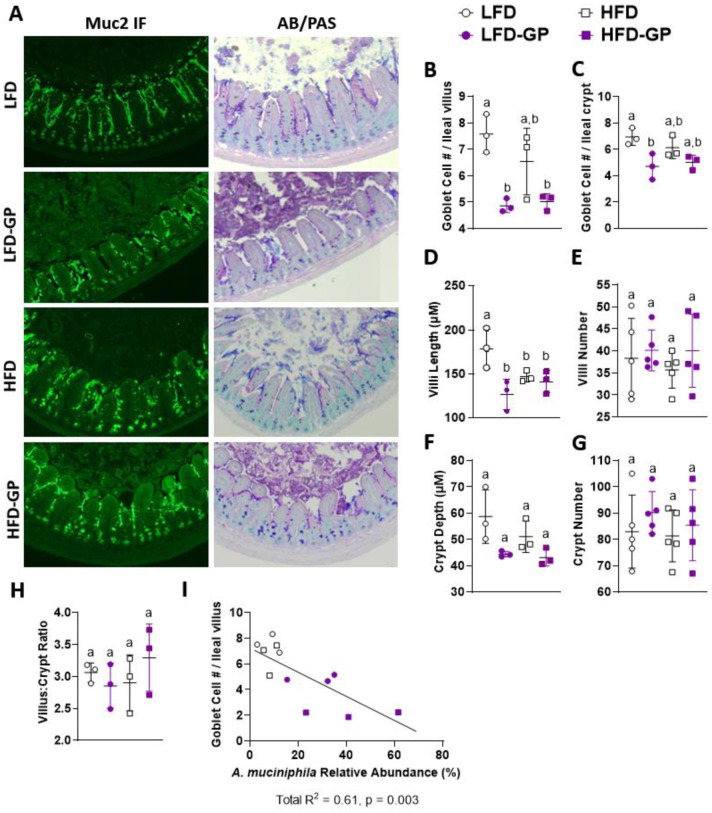
Ileum phenotypes. (A) Representative images (one mouse selected from n = 3–5 mice/group) of ileal Muc2 IF (green) and AB/PAS stains (pink, purple) at week 12 at magnification 14×. Average ileal goblet cell number (B) per villus and (C) per crypt quantified from AB/PAS stains. Average (D) villus length, (E) villi number per cross section, (F) crypt depth, (G) crypts per cross section and (H) villus-to-crypt ratio quantified from H & E stains. (I) Pearson correlation showing cecal *A. muciniphila* relative abundance verses goblet cell number per villus. Statistical significance between four groups of parametric data was determined via two-way ANOVA, followed by Holm-Sidak's multiple-comparisons test. Different letters denote statistical significance. p < 0.05 was considered significant. Each symbol denotes a single animal.

Unlike the colon, a distinct continuous mucus layer was not visible in the ileum by anti-Muc2 IF or AB/PAS staining (Figure 3A and Supplementary Figure 3). In the ileum tissue sections, Muc2 IF was visible mainly on individual villi, in crypts, and in goblet cells with some punctate spots in the ileal lumen (Figure 3A and Supplementary Figure 3). AB/PAS staining of ileal sections from LFD- and HFD-fed mice, showed punctate blue-purple staining of the mucus between villi and within goblet cells and more diffuse areas of purple in the lumen (Figure 3A and Supplementary Figure 3). The ileal tissues from the LFD-GP and HFD-GP groups showed deeper purple staining in the ileal lumen (Figure 3A and Supplementary Figure 3), suggesting increased mucus production and/or redistribution of mucus to the ileal lumen.

Goblet cell hyperplasia is a host response to infection due to the antimicrobial antibody production of these cells [60]. GP supplementation reduced the numbers of goblet cells per villus regardless of dietary fat (Figure 3B). Compared to the LFD-fed mice, goblet cell numbers per crypt were lower in the LFD-GP group, while there was a trending reduction of goblet cells per crypt in the HFD-GP group as compared to the HFD group (Figure 3C). Atrophy of villi with significant asymmetry, blunting or desquamation and crypt hyperplasia are markers of intestinal damage and dysregulation in the small intestine [61]. LFD-fed mice had significantly greater villi length than all other groups (Figure 3D). Although villi length was shorter in the LFD-GP, HFD and HFD-GP groups, the villi did not appear atrophied or damaged (Supplementary Figure 3). All diet groups had similar numbers of villi per cross section (Figure 3E), crypt depth (Figure 3F) numbers of crypts per cross section (Figure 3G) and villus-to-crypt length ratios (Figure 3H). The number of goblet cells per ileal villus was inversely proportional to the relative abundance of cecal *A. muciniphila* (Figure 3I), suggesting that GPs reduced cell differentiation to goblet cells.

### 3.4. GP supplementation reduced intestinal markers of inflammation

**Figure 4.**
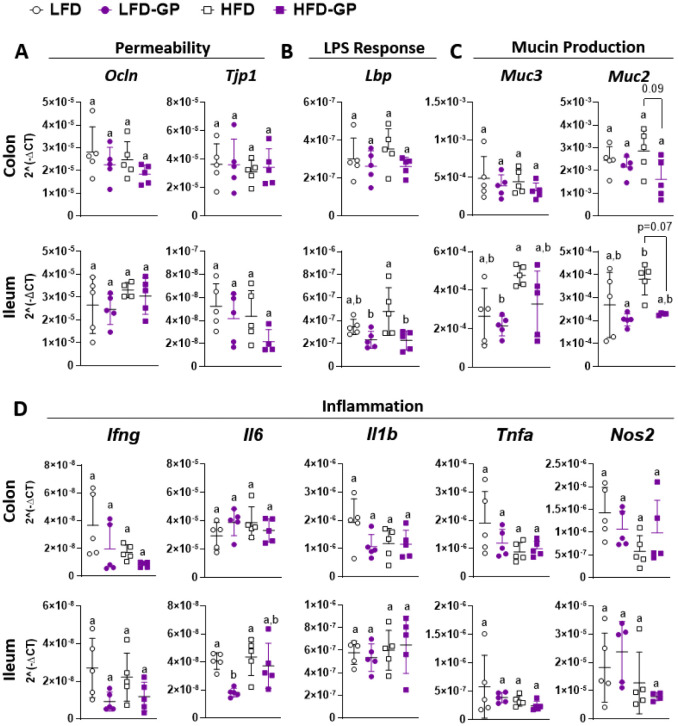
Transcriptional levels of genes important for components of gut barrier function, including regulatory genes for intestinal (A) permeability and (B) LPS response, and (C) mucin production and (D) inflammation in the colonic and ileal tissue of mice fed an LFD or HFD with or without GPs. Statistical significance of parametric data was determined via two-way ANOVA, followed by Holm-Sidak's multiple-comparisons test. Different letters denote statistical significance. p < 0.05 was considered significant. Each symbol denotes a single animal.

Markers of intestinal barrier integrity were next investigated. Tight junctions between epithelial cells are composed of multiple proteins that regulate paracellular permeability of the gut barrier. Occludin, encoded by *Ocln*, is a transmembrane protein that spans the paracellular space, while ZO-1, encoded by *Tjp1*, is a plaque protein that connects transmembrane proteins to the intracellular actomyosin ring [15]. mRNA expression levels of *Ocln* and *Tjp1* in colonic and ileal tissues were similar across the four diet groups (Figure 4A).

An HFD has been reported to induce dysbiosis, increase systemic LPS and induce chronic low-grade inflammation [20]. LBP is the initial accessory protein that is expressed to coordinate the interaction of LPS with host TLR4-MD2 receptor complexes; therefore, it is used as a surrogate marker for circulating LPS and the systemic low-grade inflammation typical of metabolic disease [21],[62],[63]. *Lbp* mRNA levels in the colon were similar among the groups (Figure 4B). In ileal tissues, mice fed an HFD showed a trending increase in *Lbp* gene expression as compared to LFD-fed mice, and this increase was suppressed in the HFD-GP group (Figure 4B), suggestive of reduced LPS presence.

LPS and other bacterial products within the intestine can stimulate mucus excretion by goblet cells [64]; therefore, we assessed the mRNA levels of *Muc2* and *Muc3* in colon and ileum tissues. Compared to the HFD group, there was a trending reduction of *Muc2*, but not *Muc3*, in the colon and ileum tissues of the HFD-GP group (Figure 4C), which may be due to the GP-induced reduction in bacterial load (Figure 1B). mRNA levels of inflammatory cytokines, *Ifng*, *Il1b*, *Tnfa* and *Nos2* were unchanged across all diet groups (Figure 4D). Compared to the LFD group, *Il6* mRNA was reduced in the LFD-GP group (Figure 4D). The HFD and HFD-GP groups did not show differences in inflammatory gene expression (Figure 4D).

Dietary polyphenols may be perceived by the body as xenobiotics which activate Phase II enzymes to conjugate polyphenols with glucuronate, sulfate, acetate or glutathione moieties to reduce their bioactivity and increase their water solubility for ease of excretion [65],[66]. Induction of the Phase II metabolism prevents injury by plant-derived xenobiotics [65],[67]. Hepatic serum ALT, is commonly used as a biomarker of hepatocellular necrosis induced by xenobiotics or an HFD [66],[68]. Levels of serum ALT were within the expected range of 4–36 IU/L and were not significantly increased by dietary fat (HFD vs LFD, p = 0.45) or by GP supplementation on an LFD (LFD vs LFD-GP, p = 0.07) or HFD (HFD vs HFD-GP, p = 0.31) (Supplementary Figure 4), suggesting that no hepatic injury occurred due to diet or GP supplementation. [69]

## 4. Discussion

Several studies have shown that GPs attenuate diet-induced inflammation [27],[43],[44],[70]. Supplementation of male C57BL6/j mice with 1% w/w GP diets reduced levels of LPS, inflammatory cytokines, IL-6 and TNFα in circulation [27]. GPs were also found to increase the expression of intestinal TJP *Ocln*, improve glucose metabolism and lower levels of intestinal inflammatory cytokines and ROS accumulation [27],[43],[70]. Disruption to the gut barrier has been implicated behind the development of diet-induced metabolic endotoxemia and insulin resistance [20].

The anti-inflammatory effects of GPs occurred in association with increased abundance of *A. muciniphila* and significant modulations to the gut microbial community [27]. Previous in vitro experiments showed that GPs and pure PACs inhibited the growth of *A. muciniphila* [42]; therefore, the observed bloom in this microbe is unlikely due to a direct growth-promoting effect of GPs. Rather, the GP-induced proliferation of *A. muciniphila* may be due to GP-mediated suppression of competing microbes in the gut [42]. Similarly, an antibiotic cocktail of doxycycline, hydroxychloroquine, piperacllin/tazobactam and teicoplanin has been shown to reduce total bacterial load while promoting a bloom in *A. muciniphila* [71]. Not all antibiotics induce such a bloom in *A. muciniphila*, as a streptomycin and bacitracin cocktail promoted the growth of *Turicibacter* or *Staphylococcus* in murine gut [72]. We hypothesize that GPs have selective antibacterial effects that result in the suppression of competing microbes, which affords *A. muciniphila* with greater opportunity for proliferation.

Oral administration of *A. muciniphila* has been reported to increase colon mucus thickness in HFD-fed mice [26]; therefore, our initial hypothesis was that the GP-induced bloom of *A. muciniphila* contributed to reduced serum LPS levels by stimulating an increase in mucus layer thickness. In contrast, we observed that GP-supplemented mice had a thinner colonic mucus layer (Figure 2A and B), increased fecal mucus concentrations (Figure 2D), reduced ileal goblet cell numbers (Figure 3B and C) and downregulated *Muc2* expression (Figure 4C), regardless of dietary fat.

Other studies have indicated that dietary polyphenol supplementation increased fecal mucus [73],[74]; however, in these studies, measurements of mucus layer thickness were not performed, and it was not determined whether polyphenols induced mucus production or a redistribution of mucus into the fecal pellet. The polyphenol EGCG, which is abundant in green tea, was found to aggregate salivary mucins and reduce mucin networking due to its protein-precipitating properties [75]. As our GP extract contained type-B PAC dimers, trimers, tetramers and pentamers [42], such polyphenol-mucin aggregates may contribute to the presence of punctate or rod-like Muc2-staining particles within the digesta of GP-supplemented mice, as well as the reduced appearance of a compact mucus wall (Figure 2A).

The GP-induced bloom of *A. muciniphila* (Figure 1A and C) may explain the decreased mucus layer in the colon and redistribution of mucus to the fecal pellet or digesta (Figure 2D and Supplementary Figures 2 and 3). There was a significant negative correlation between the percent relative abundance of mucin-degrading *A. muciniphila* and mucus layer thickness based on GP supplementation, indicating that the thinner mucus layer in GP-supplemented mice was driven by the GP-induced increase in *A. muciniphila* abundance (Figure 2G). Everard et al. showed that HFD-fed mice administered live *A. muciniphila* had increased mucus thickness compared to HFD-fed controls; however, unlike GP supplementation [27],[42],[43],[76], the *A. muciniphila* treatment did not significantly change the gut microbial community structure or reduce bacterial load [26]. Broad spectrum antibiotic treatments promote a bloom in *A. muciniphila* [71], which was further associated with protection from diabetes in NOD mice [77]. Thus, GPs may have caused mucus thinning both due to increases in *A. muciniphila* and its antimicrobial effects on select bacteria.

The mucus layer in the colon is produced in response to bacteria and bacterial products. Germ-free mice deficient in gut bacteria, were shown to have a very thin colonic mucus layer, but, after LPS or peptidoglycan administration, their colonic mucus layer thickness was similar to conventional mice [78], indicating that bacterial proteins are a major stimuli for intestinal mucus production. Specific species of the Firmicutes phyla have been shown to increase expression of mucins [79],[80]; therefore, GPs may selectively inhibit Firmicutes bacteria, which promote mucin expression [27]. Other studies have indicated a thicker mucus layer [81] and an altered mucin glycoprotein composition with larger and more complex mucins in conventional mice as compared to germ-free mice [82]. Cecal goblet mucin has also exhibited stronger reactivity to lectin-dependent staining in conventional mice than germ-free mice, supporting altered mucus composition [83]. Additionally, conventional mice have been observed to have more and larger goblet cells [83], providing further evidence for a promotion of mucus production by gut bacteria.

Supplementing germ-free or conventional mice with PAC-rich pea seed coat extract increased fecal mucin levels [74], suggesting that PACs directly increase mucus production rather than suppressing the production via alterations to gut bacteria. Additionally, mucin genes (*Muc1–4*) were counterintuitively expressed at higher levels in germ-free mice when compared to conventional mice [84]; however, it is not known how the fecal mucin level or mucus layer thickness was affected. Furthermore, antibiotic treatment has been shown to increase [72] and decrease [85] colonic mucus layer thickness.

While these germ-free and antibiotic studies highlight the influence of a bacterial community on the mucus layer, the direct relation between gut bacteria and the mucus layer is not yet delineated. Additionally, germ-free mice are biologically different from conventional mice, for example, with respect to immune system maturation, which may confound comparison. Studies that consider the gut microbiota, host transcription of mucin genes, fecal mucin concentrations and histochemistry of the mucus layer together are required to gain insight into the effects of bacteria on the mucus layer in the context of different treatments. Similar to germ-free mice, GP-supplemented mice had a lower total bacterial load, a thinner colonic mucus layer and fewer goblet cells in ileal tissues. Unlike germ-free mice, GP-supplemented mice had reduced transcription of *Muc2* compared to unsupplemented control groups (Figure 4C). The observed intestinal phenotypes may therefore be at least partly due to antibacterial effects of the GPs and lower numbers of microbes that normally stimulate host mucus production.

A thinner mucus layer is indicative of gut barrier disruption; however, GP-supplemented mice did not present with markers of inflammation in intestinal tissue. Furthermore, IL-6 expression was reduced in the ileal tissue of supplemented LFD-fed mice (Figure 4D). Taken together, we suspect that the antibacterial properties of GPs reduced the need for a protective colonic mucus layer and high numbers of goblet cells. The necessity of the mucus layer may be increased in the presence of a higher bacterial load of pathogenic species. With the lower bacterial load, GP-supplementation likely promotes an intestinal environment with less need for colonic mucus for protection.

## 5. Conclusions

These data provide a novel perspective on the role of the intestinal mucus layer, suggesting that the mucus layer may not always be an integral host response for maintaining gut barrier integrity. We propose that the function of the gut mucus layer is conditional on the microbial community. Further studies are warranted to investigate how dietary polyphenols influence the mucus layer, as well as the physiological implications.
