# Supplementary material for: Impact of grape polyphenols on Akkermansia muciniphila and the gut barrier
Source: AIMS Microbiol. 2022 Dec 22;8(4):544–65. doi: 10.3934/microbiol.2022035 (PMC9834079; doi:10.3934/microbiol.2022035)
Supplement: Supplementary file 1 [file microbiol-08-04-035-s001.pdf]

Microbiology

AIMS Microbiology, 8(4): 544–565.
DOI: 10.3934/microbiol.2022035
Received: 23 September 2022
Revised: 16 December 2022
Accepted: 17 December 2022
Published: 22 December 2022

http://www.aimspress.com/journal/microbiology

Research Article

Impact of grape polyphenols on *Akkermansia muciniphila* and the gut barrier

Esther Mezhibovsky [1,2], Yue Wu [1], Fiona G. Bawagan [1], Kevin M. Tveter [1], Samantha Szeto [1] and Diana Roopchand[1,*]

[1] Rutgers, The State University of New Jersey, Department of Food Science, Institute for Food Nutrition and Health [Center for Microbiome, Nutrition and Health & Rutgers Center for Lipid Research], 61 Dudley Road, New Brunswick, NJ 08901, USA

[2] Rutgers, The State University of New Jersey, Department of Nutritional Sciences Graduate Program, New Brunswick, NJ 08901, USA

* **Correspondence:** Email: roopchand@sebs.rutgers.edu; Tel: 8489320248.

Supplementary

Supplementary Table 1. Nutritional evaluation of grape polyphenol-soy protein isolate (GP-SPI) complex and SPI (per 100 g)

assay	SPI	GP-SPI
Calories	386	388
Calories from fat	29	34
Ash %	3.9	4.0
Moisture %	4.6	3.75
Carbohydrates %	0	33.6
Total Dietary Fiber	< 0.1	24.9
Protein %	89.3	54.9
Total Fat %	3.24	3.77
Sugars %		
Glucose	< 0.1	0.84
Fructose	< 0.1	1.2
Sucrose	< 0.1	< 0.1
Total Sugars %	0	2.5

Supplementary Table 2. Nutritional Composition of Diets HFD with 10% SPI (HFD).

		HFD with 10% GP-SPI (HFD-GP)	LFD with 10% SPI (LFD)	LFD with 10% GP-SPI (LFD-GP)
Ingredient (g/kg)				
Casein	158.2	157.6	93.5	98.9
Soy protein, Supro 661	0	39.4	0	28.9
DL- methionine	3.9	3.9	2.84	2.84
GP-SPI	0	100	0	100
SPI	100	0	100	0
Corn starch	6.7	0	484	477.5
Maltodextrin 10	162.1	161.5	118.5	118.5
Sucrose	80.5	80.2	58.9	58.9
Dextrose	0.8	0	0.85	0
Fructose	1.2	0	1.23	0
Cellulose	65.0	40.0	47.4	22.6
Lard	318.0	316.5	19.0	19.0
Soybean oil	30.3	28.0	21.7	19.7
Mineral mix S10026	13.0	12.9	9.5	9.5
Dicalcium phosphate	17.0	16.8	12.3	12.3
Calcium carbonate	7.1	7.1	5.2	5.2
Potassium citrate	21.4	21.3	15.6	15.6

Continued on next page

		HFD with 10% GP-SPI (HFD-GP)	LFD with 10% SPI (LFD)	LFD with 10% GP-SPI (LFD-GP)
Ingredient (g/kg)				
Vitamin Mix V10001	13.0	12.9	9.5	9.5
Choline bitartrate	2.6	2.6	1.9	1.9
Red dye #40, FD&C	0.065	0	0	0.04
Blue dye #1, FD&C	0	0	0.04	0.01
Yellow dye #5, FD&C	0	0.065	0.01	0
Total (g)	1000	1000	1000	1000
Gram%				
Protein	23.2	23.1	17.0	17.0
Carbohydrate	26.4	25.6	67.3	66.6
Dextrose	0.1	0.1	0.1	0.1
Sucrose	9.3	9.3	6.9	6.8
Fructose	0.1	0.1	0.1	0.1
Maltodextrin	16.2	16.1	11.8	11.8
Corn starch	0.67	0	48.4	47.8
Fat	35.3	35.2	4.5	4.5
Fiber	6.5	6.5	4.8	4.7
SPI	10.0	0	10	0
GP-SPI	0	10	0	10
kcal (%)				
Protein	18	18	18	18
Carbohydrate	20	20	71	71
Fat	62	62	11	11
Total	100	100	100	100
kJ/g (calculated)	21.64	21.56	15.87	15.81
kCal/g (calculated)	5.17	5.15	3.79	3.78

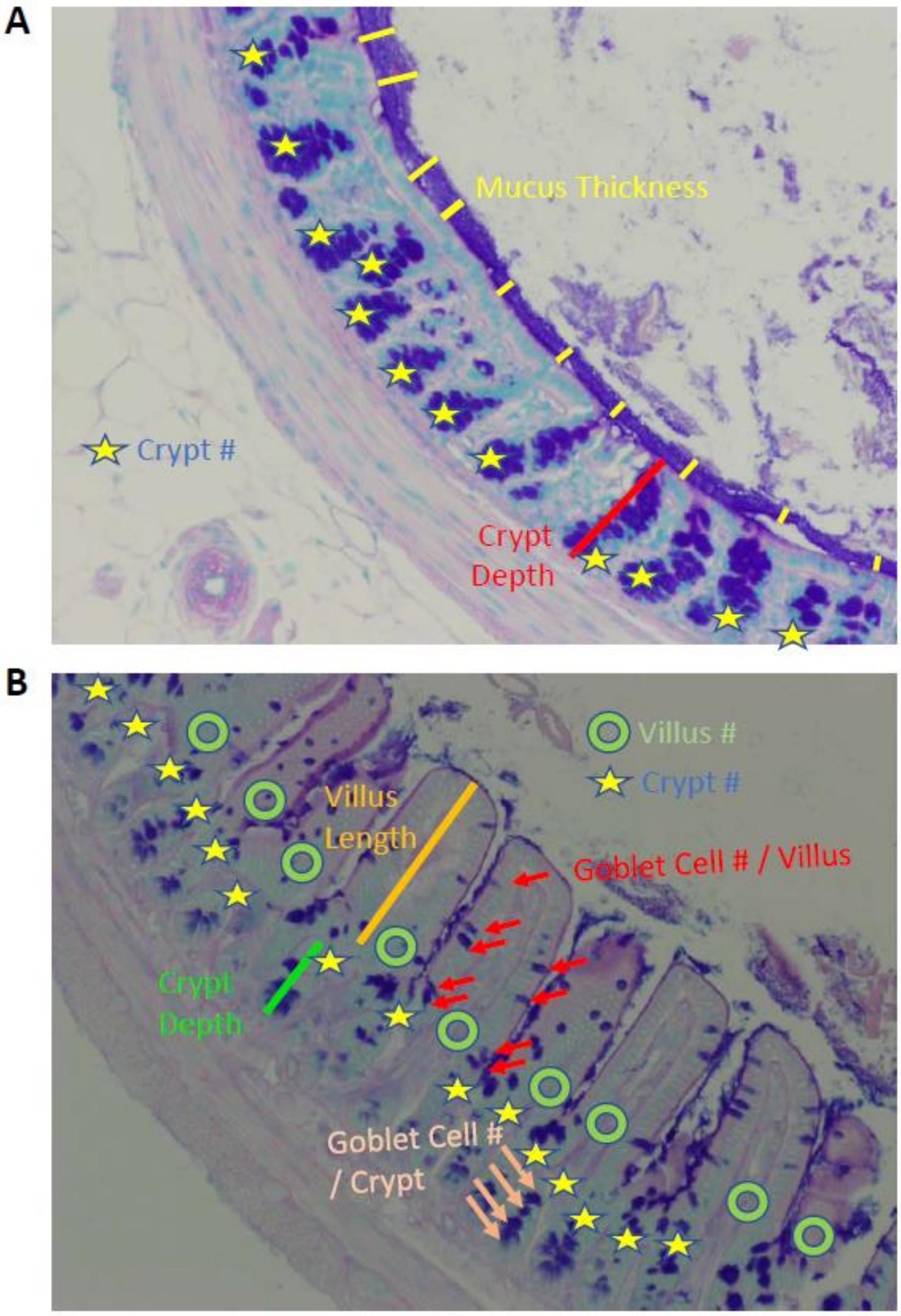

Supplementary Figure 1. Labeled sections displaying **(A)** colon image used for measuring colonic mucus thickness, crypt depth and number, and**(B)** ileum images used for ileal villus length, villus number, crypt depth, goblet cell number/villus, and goblet cell number/crypt. Magnification: 14x.

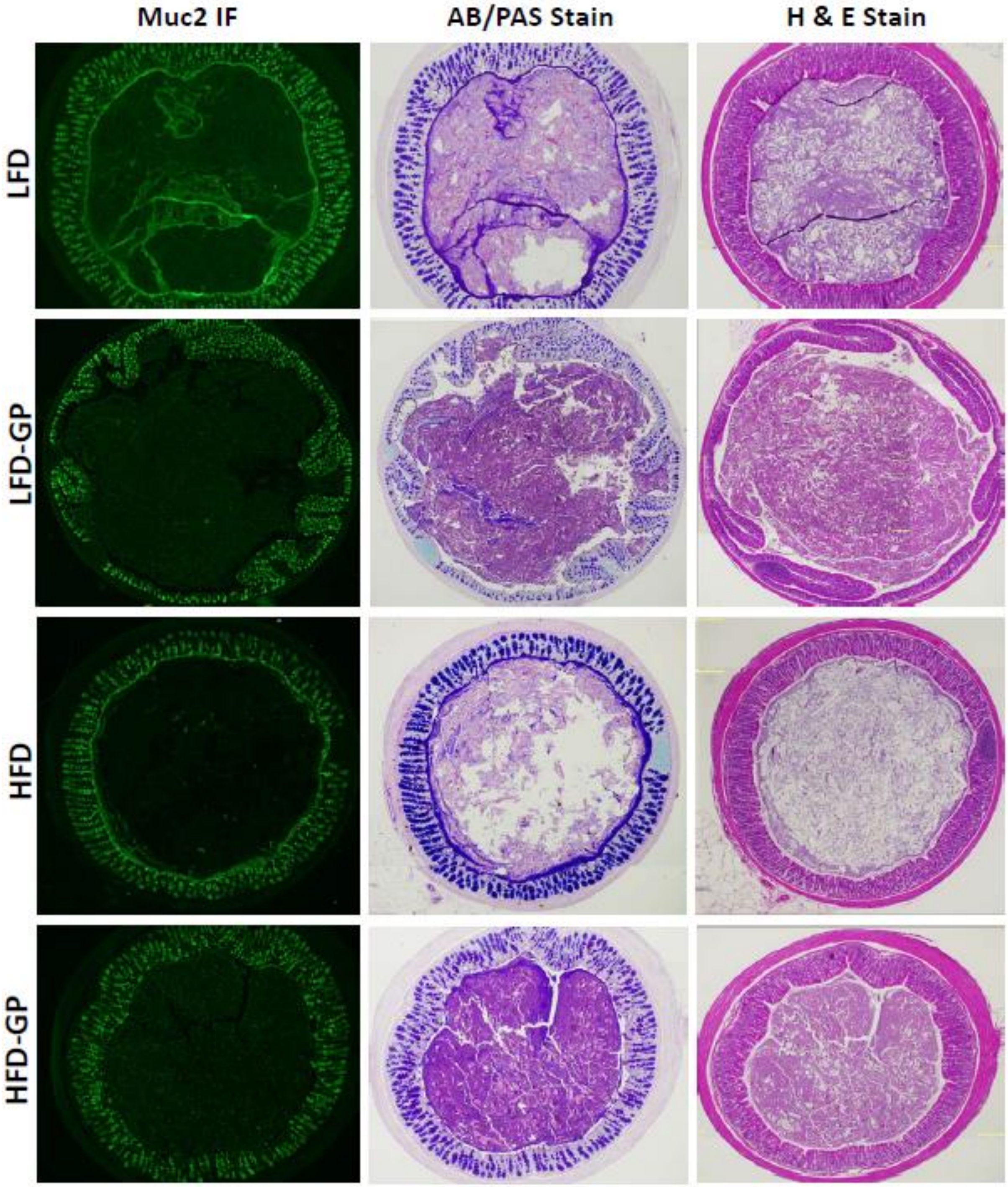

Supplementary Figure 2. Representative images of whole colonic cross-sections (one mouse selected from n= 3 –5 mice/group) subjected to Muc2 IF (green) and AB/PAS (purple, pink) staining with digesta intact from LFD, LFD-GP, HFD, HFD-GP mice after

12 weeks on diet.

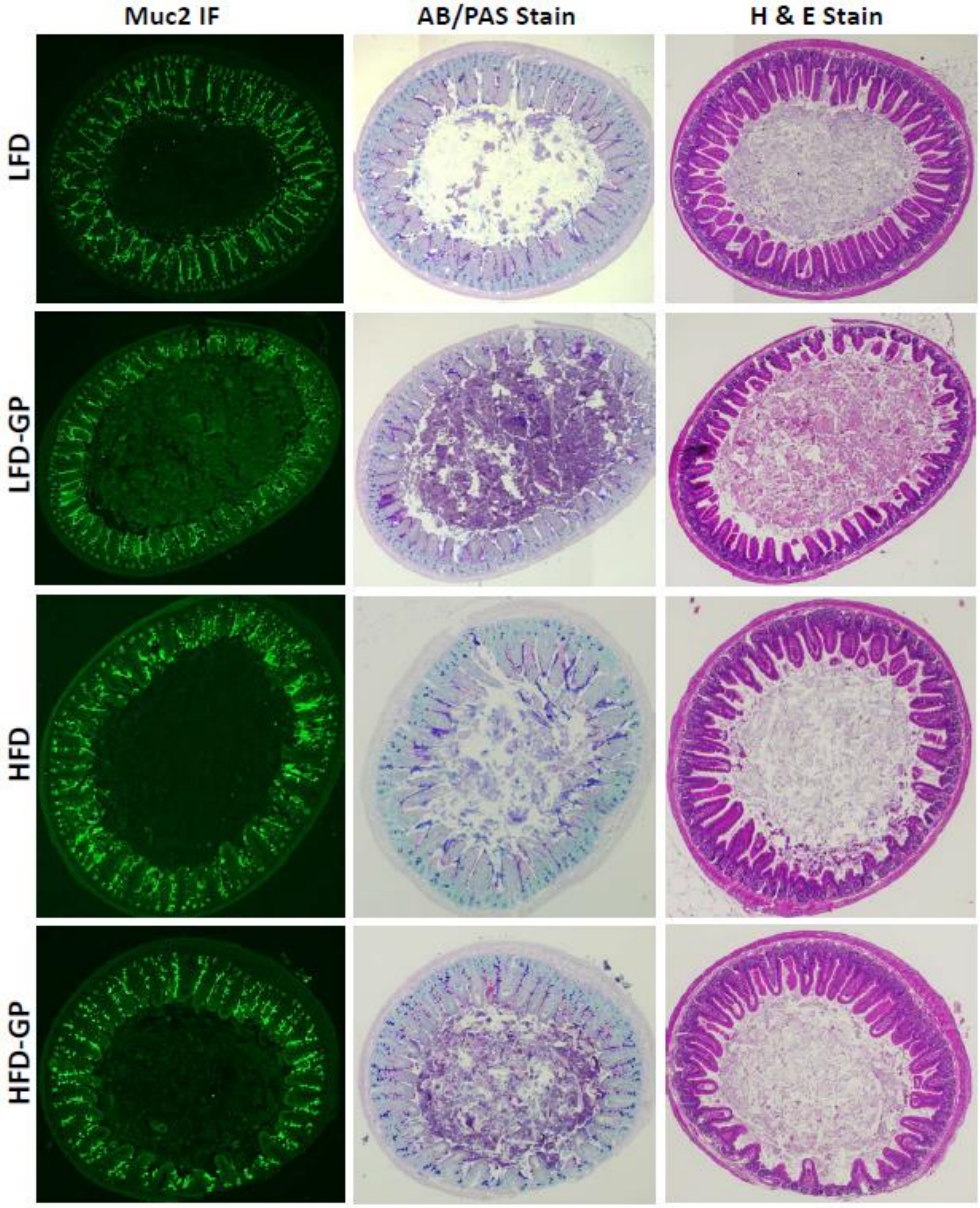

Supplementary Figure 3. Representative images (one mouse selected from n= 3 –5 mice/group) of whole ileal cross-sections subjected to Muc2 IF (green) and AB/PAS (purple, pink) staining with digesta intact from LFD, LFD-GP, HFD, HFD-GP mice after 12 weeks on diet.

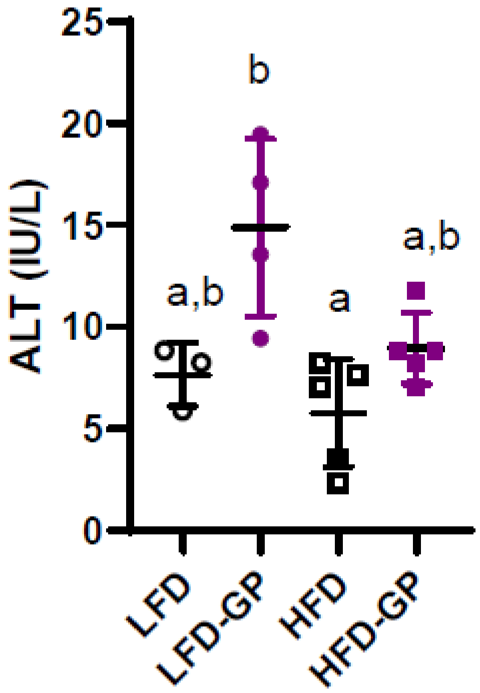

Supplementary Figure 4. Concentration of alanine transaminase (ALT) in cardiac serum collected after 12 weeks of the diet-intervention.
